# *MDM2* promoter SNP285 and SNP309; phylogeny and impact on cancer risk

**DOI:** 10.18632/oncotarget.243

**Published:** 2011-03-23

**Authors:** Stian Knappskog, Per E. Lønning

**Affiliations:** ^1^ Section of Oncology, Institute of Medicine, University of Bergen, 5020 Bergen, Norway; ^2^ Department of Oncology, Haukeland University Hospital, 5021 Bergen, Norway

**Keywords:** MDM2, p53, polymorphism, breast cancer, ovarian cancer, evolutionary selection

## Abstract

MDM2 plays a key role to physiological processes like growth arrest, senescence and apoptosis. It binds to and inhibits key proteins like p53 and the RB protein, and *MDM2* amplification as well as protein overexpression without amplification is seen in many solid tumors. An *MDM2* promoter polymorphism (SNP309T>G) has been found associated with enhanced Sp1 transcription factor binding and elevated MDM2 transcription. While 309G has been found associated with elevated cancer risk and young age at diagnosis of different cancers, results in Caucasians have been at variance. Recently, we reported a second polymorphism (SNP285G>C) located on the 309G allele. The 285C/309G haplotype accounts for about 12% of all 309G alleles among Norwegians, Dutch and British habitants. Assessing Sp1 binding to the *MDM2* promoter using surface plasmon resonance technology, we found SNP309G to enhance Sp1 binding by 22% while SNP285C reduced Sp1 binding by 51%. SNP285C reduced the risk of breast cancer and ovarian cancer among 309TG/309GG carriers by 21 and 26%, respectively, but in particular the risk of ovarian cancer among 309TG heterozygotes (reduction by 37%). The fact that the 285C/309G haplotype accounted for only 1.9% of all 309G alleles among Finns and was absent in Chinese indicate 285C to be a young polymorphism.

Murine Double Minute Clone 2 (MDM2) plays a key role in the regulation of cell cycle and apoptosis. The *MDM2* gene was originally discovered and cloned from a spontaneously transformed BALB/c 3T3 mouse cell line [1], and it was later shown to act as an oncogene in nude mice [2]. The MDM2 protein acts as a p53 antagonist and is linked to p53 in a feedback-loop where p53 enhances MDM2 transcription in response to genotoxic stress, whereas MDM2 binds to p53 and directs it for proteosomal degradation through ubiquitinylation [3, 4]. The vital importance of this MDM2 — p53 interaction is underlined by the fact that although MDM2 null mice uniformly die at an early embryonic stage, MDM2/p53 null double knock-outs are live born with no obvious developmental defects [5, 6].

MDM2, in addition, inactivates the retinoblastoma protein (pRB) by protein binding [7], preventing pRB from binding and inactivating E2F1. Further, it directly interacts with and stimulates E2F1 [8], a transcription factor with complex functions, both able to promote cell cycle progression through induction of genes coding for e.g DNA polymerase and cyclin E but also apoptosis through induction of genes coding for e.g. APAF1 and caspases [9]. The fact that MDM2 acts as a key regulator of both the p53 and the pRb functional pathways points to *MDM2* as a “master” gene potentially governing the balance between cellular processes like continued growth, growth arrest, senescence and apoptosis.

## SOMATIC ALTERATIONS OF *MDM2* IN MALIGNANT TUMORS

So far, very few somatic mutations in the *MDM2* gene has been identified. The gene however, has been found amplified in many tumour forms [reviewed in 10]. In addition, several tumors reveal elevated staining for the MDM2 protein despite harbouring a normal gene copy number. Thus, several mechanisms apart from *MDM2* gene amplification has been suggested to influence MDM2 levels and activity. These include enhanced translation through increased activity of BCR/ABL [11-13] and expression of a variety of alternatively or aberrantly spliced *MDM2* mRNA variants [14]. While splice variants have been reported to inhibit the function of wild-type MDM2 through dimerization [15, 16], the full function of these alternatively spliced mRNAs remains to be elucidated. Recently, another alternative mechanism of MDM2 upregulation, disturbing proper function of the p53 pathway, was reported: Inuzuka and co-workers demonstrated that MDM2 is phosphorylated by Casein Kinase I (CKI), marking the MDM2 molecule for degradation via the SCF^β-TRCP^ Ubiquitin ligase [17]. This has been suggested as a potential mechanism of MDM2 upregulation in cases where CKI and / or β-TRCP are inactivated [18], a hypothesis that is supported by the findings of β-TRCP-deletions in several tumor forms [18-20].

## GERM LINE ALTERATIONS OF *MDM2* FUNCTION

Despite its role as a proto-oncogene, so far, neither *MDM2* germ line mutations nor increased gene copy number at the germ line level has been identified in cancer prone families. Thus, one may speculate whether loss of MDM2 function through deleterious mutations may be incompatible with life, resembling the effect in *MDM2* null mice.

**Figure 1:**
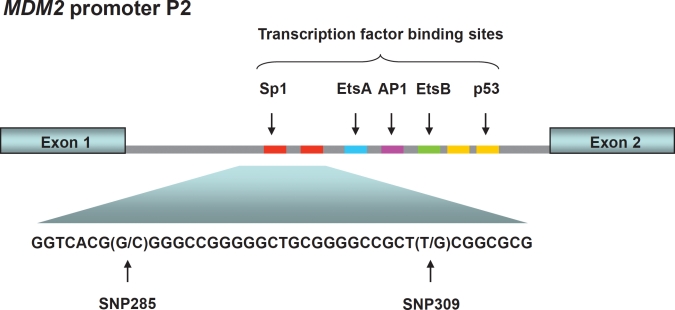
*MDM2* promoter P2 This promoter is located between the exons 1 and 2 of the *MDM2* gene and is induced upon cellular stress. Promoter P2 harbors binding sites for among others p53 and Sp1. SNP285 and SNP309 are both located within the latter binding sites.

The *MDM2* gene contains two main promoters; the P1 located upstream of exon 1, and the intronic P2 promoter, located between exon 1 and exon 2. P1 is considered responsible for regulating *MDM2* expression levels in the “non-stressed” setting. This promoter contains a binding site for PTEN, which has been shown to suppress MDM2 expression. The P2 promoter is considered to be more inducible and regulates MDM2 level in response to cellular stress and stimulation by different ligands including p53, Sp1 and the estrogen receptor (Figure 1) [21, 22].

In 2004, the group lead by Arnold Levine identified a polymorphism SNP309 (T>G; rs2279744) in the P2 promoter [21]. The G-allele is found across all ethnic groups albeit at a variable frequency (~10% of *MDM2* alleles among African Americans, 40% in Caucasians and 50% in Asians) [23]. It was found to increase the expression levels of *MDM2*through enhancing the binding of the Sp1 transcription factor. The G-allele was linked to early age at cancer diagnosis among individuals with Li-Fraumeni syndrome (carrying germline *TP53* mutations) and an early age at diagnosis of soft tissue sarcomas, large B-cell lymphomas and colorectal cancers in women not diagnosed with any cancer predisposing germline mutations [21, 24]. In addition, an early diagnosis of estrogen receptor rich (defined as >50% of cells revealing positive ER staining) but not receptor-poor breast cancer was recorded [21]. Although these data are strongly supported by the finding that *MDM2*^SNP309G/G^mice are more tumor prone than *MDM2*^SNP309T/T^ mice [25], case control studies across many tumor forms and ethnic groups have provided conflicting results regarding the role of the SNP309G-allele as a cancer risk factor in humans [23, 26]. Notably, so far most of the studies linking the SNP309G variant allele to enhanced cancer risk or a young age at diagnosis have been performed on Asian or Ashkenazi Jewish populations [23, 26]. In contrast, the majority of studies performed in Caucasians are negative [23, 26]. In addition to SNP309, several other SNPs have also been reported both in the promoter P2 area and the rest of the *MDM2* gene. Notably, in the three most thoroughly studied ethnic groups (Caucasians, African Americans and Ashkenazi Jews) the variability in other polymorphic sites is much higher for the SNP309T-allele than for the G-allele [27]. Taken together with the relatively high frequency of SNP309G, this may indicate a positive selection pressure for the G-variant. Notably, the reported polymorphisms in the *MDM2* gene are restricted to either SNP309T or G, indicating that they are of younger origin than the 309 variation. To this day, potential biological function of these polymorphisms remains to be elucidated.

## SNP285C REDUCES THE RISK OF BREAST AND OVARIAN CANCER

**Figure 2:**
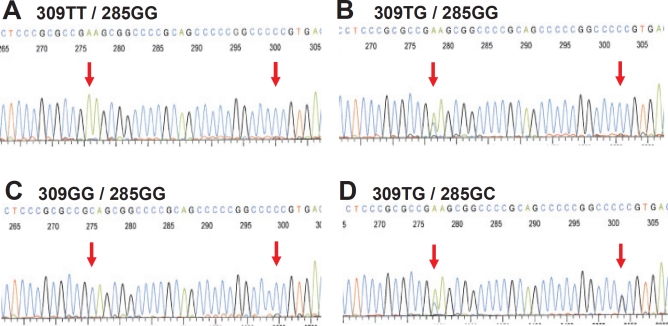
SNP285 and SNP309 genotypes Electropherograms showing the four different genotypes of the Sp1-binding elements of MDM2 promoter P2 commonly observed among Caucasians. (Electropherograms display sequence as reverse complementary to the open reading frame of MDM2)

Studying the potential effects of *MDM2* promoter SNP's on breast cancer therapy, outcome and prognosis, we, concomitantly with a Scottish group [28], discovered a second promoter P2 polymorphism, 285G>C, located 24 bps from SNP309 (Figure 2) [29]. The C-variant of SNP285 is located on the SNP309G allele forming a distinct SNP285C/309G haplotype. Notably, SNP285C was found at a similar frequency (average of 7.8% of individuals) in different Western populations (Dutch, British, Norwegian) but was absent from Asians (Chinese). Interestingly, the frequency of this polymorphism among Finnish individuals living in the Helsinki area (1.6%), was significantly lower compared to the frequency observed among other Western Europeans. Potential interpretations of this finding is discussed below.

**Figure 3:**
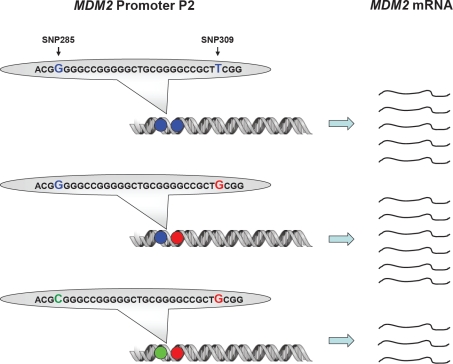
Impact of SNP309 and SNP285 on MDM2 transcription Presence of the SNP309G-variant (red) enhances the binding strength between MDM2 promoter P2 and the transcription factor Sp1, thus, leading to increased MDM2 transcription as compared to the “wild-type” SNP309T-variant. The effect of the SNP285C-variant (green) is the opposite, reducing the binding strength for Sp1, thus, also reducing the level of MDM2 transcription as compared to the “wild-type” SNP285G-variant. The effect of SNP285C is stronger than SNP309G, thus, presence of the SNP285C/309G haplotype leads to reduced MDM2 transcription as compared to the “wild-type” SNP285G/309T haplotype.

Using the highly sensitive surface Plasmon resonance technology (SPR), we confirmed the previous findings of Bond et al [21] that the SNP309G-allele had a stronger binding affinity to the Sp1 transcription factor as compared to the SNP309T-allele (22% increase in binding strength). In contrast, SNP285C significantly reduced Sp1-binding to the *MDM2* promoter (51% decreased binding strength). Importantly, the combined SNP285C / SNP309G haplotype (which accounts for about 12% of all 309G alleles in Caucasians) had a reduced affinity towards Sp1 as compared to the “wild-type” SNP285G/309T haplotype (>10% decreased binding strength) [29]. While secondary mutations antagonizing a primary mutation in *BRCA2* through excision of the mutated area has been shown to revert platinum drug sensitivity in breast as well as ovarian carcinomas [30, 31], to the best of our knowledge, secondary SNP's antagonizing the effect of a previous SNP on the same gene, have not previously been reported (Figure 3).

In line with the finding of reduced binding strength to the Sp1 transcription factor and, thereby, reduced transcription, we found the presence of SNP285C to be associated with significantly lowered risk of both breast and ovarian cancer. Among individuals potentially harbouring the SNP285C variant (carriers of SNP309G) we found a 21% reduced risk of breast cancer among individuals carrying SNP285C as compared to those homozygous for SNP285G. Regarding ovarian cancer risk, the effect was even stronger. Here, the presence of a SNP285 allele lead to a reduction in risk of disease of 26%. Among individuals carrying the SNP309TG heterozygous genotype, SNP285C reduced the risk of ovarian cancer by 37%, while no effect was observed in SNP309GG homozygotes. In contrast, the effect of 285C on breast cancer risk was most profound in SNP309GG homozygotes (reduction of 45%), with a small non-significant reduction of 9% in SNP309TG heterozygotes.

The data for ovarian cancer patients fits well with the *in vitro* data and a hypothesis that one SNP285C may counteract and neutralize the effect of one SNP309G, but not two SNP309Gs. The results from the breast cancer cohorts may seem somewhat harder to understand; notably, it is well known from other genes that heterozygote carriers of a genetic alteration may be at risk of different diseases than the homozygotes [32].

The finding that the SNP285C/309G haplotype accounted for about 12% of all SNP309G alleles among Caucasians may be of importance explaining the potential difference regarding the effect of SNP309 status on cancer risk among Caucasians versus Asians [23, 26]. While studies on Asian populations may represent the “true” effect of SNP309G, studies on Caucasians may need to be corrected for the presence on of the SNP309G-counteracting SNP285C in order to unmask the real effect of SNP309G in these populations.

Importantly, so far the potential effect of SNP285C on the risk of malignancies other than breast and ovarian cancer has not been explored. Considering other risk modulating factors associated with breast and ovarian cancers, notably both diseases are found at high incidence among individuals carrying pathogenic *BRCA1*and *BRCA2* germline mutations. However, this does not mean that breast and ovarian cancer have a particular and common etiology indicating that the effect of SNP285C may be restricted to these two malignancies. For example estrogen exposition is a major risk factor associated with breast but not ovarian cancer [33]. Thus, it may well be that SNP285C modulate the risk of several other malignancies in addition to breast and ovarian cancer

## EVOLUTIONARY SELECTION OF *MDM2* SNPS

Examining *MDM2* haplotype status across ethnic groups, Atwal and colleagues recorded multiple polymorphisms with an ethnic diversity between Caucasians (general population), Ashkenazi Jews and American-Africans [27]. They found a single SNP309G haplotype in Africans and Caucasians, but two additional 309G-linked polymorphisms in Ashkenazi's. In contrast, multiple SNPs were recorded on the T-allele, mostly located outside the promoter area and outside the exons. However, except for the SNP309G and now SNP285C, the potential functional role of the different polymorphisms have not been determined. Thus, we may not at this stage speculate on potential reasons for evolutionary selection (if any) for these additional SNP's.

The variable distribution of SNP309G and, now, in particular the SNP285C, across ethnic groups may lead to speculations on the phylogenetic selection of these particular *MDM2* variants. Considering the key role of MDM2 influencing the p53 as well as retinoblastoma pathway, naturally a fine tuning of its biological activity must be of critical importance, as documented by the influence of SNP285/309 haplotype status on cancer risk. These observations raise further important questions. Considering SNP309G, this variant enhances MDM2 transcription, subsequently antagonising p53 function. The fact that the SNP309G genotype has been found associated with increased cancer risk as well as an early cancer diagnosis [21, 26] may suggest a negative selection for this variant. However, with a few exceptions solid tumours in general are diagnosed after the age of child-birth. Thus, early death related to malignant disease is unlikely to explain evolutionary selection of the SNP309 variants.

Regarding the p53 Pro72Arg polymorphism, the Arginine variant has been shown to reveal stronger pro-apoptotic effects as compared to the Proline allele [34]. In Africa, the p53 proline variant becomes more frequent approaching the equatorial line [35], and Atwal et al postulated that a high frequency of *MDM2* SNP309T among Africans may act in concert balancing a weaker p53 codon 72 proline variant [27]. So far however, we know the frequency of the *MDM2* SNP309T and G variants among American Africans only; thus, to confirm such a hypothesis, assessing the SNP309 status across different geographical and ethnic groups in Africa is warranted. Further, studies on the distribution of SNP309G across different areas in Africa as well as in Asia may add important information with respect to potential mechanisms selecting for this polymorphism in general. While Fang et al, in two small studies, reported *MDM2*SNP309G to be associated with increased risk of missed abortion among Han Chinese [36, 37], this finding needs to be confirmed in larger studies involving other ethnic groups as well.

**Figure 4:**
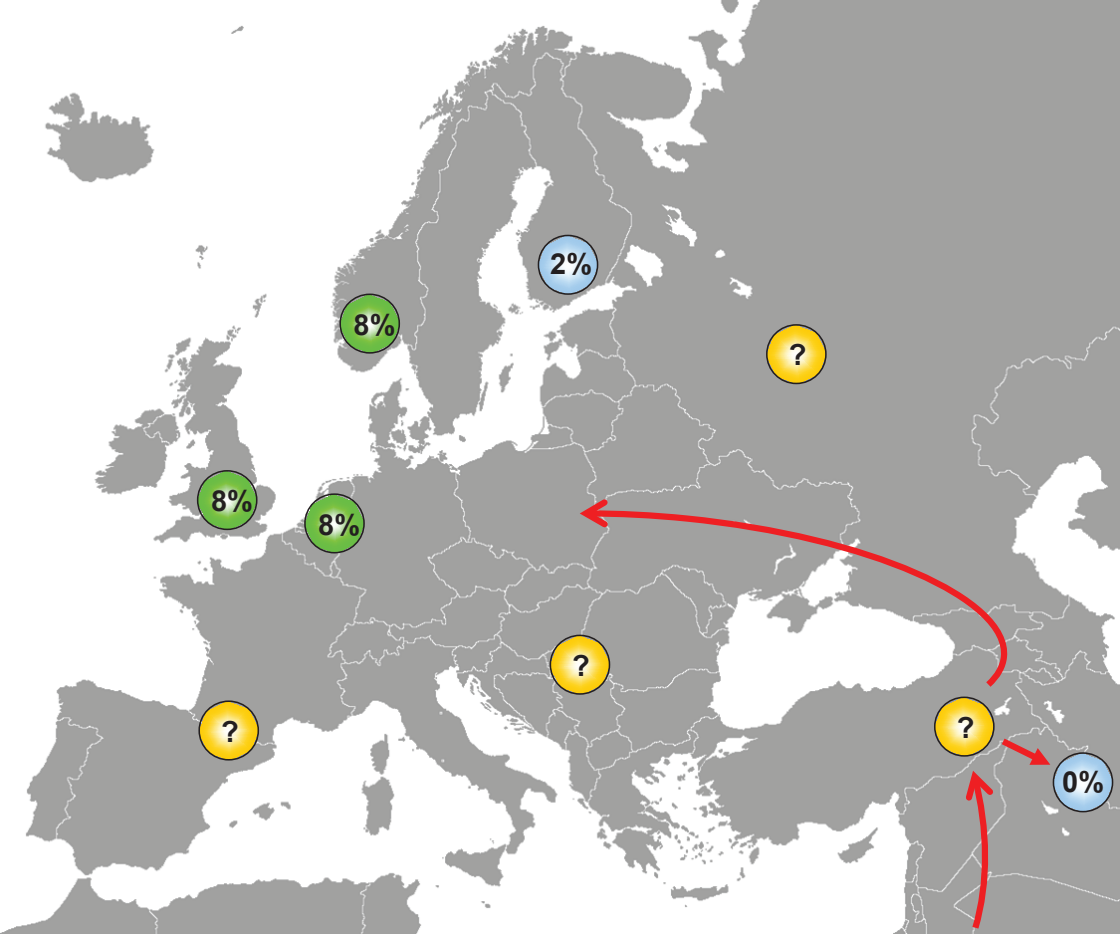
Distribution of SNP285C among different ethnic groups SNP285 is observed among ~8% of British, Dutch and Norwegians and in ~2% of Finns, while it is absent in Chinese. The presence of SNP285C only among Caucasians indicates that this polymorphism has originated after the separation of Caucasians and Asians approximately 60,000 years ago, but studies on other European populations are warranted to shed light on the origin of SNP285C. Most likely, SNP285C should be absent in all ethnic groups in the Middle-East, but this has yet to be confirmed.

We do not know the phylogenetic age of either the SNP309G or the SNP285C polymorphism, but some assumptions may be made. The fact that SNP309G exists in all ethnic groups (albeit at different incidence) indicates this variant to be an ancient polymorphism, while the fact that SNP285C locates to the SNP309G allele and is absent in Han Chinese, indicates SNP285C to be a young polymorphism, appearing in Caucasians after the “out of Africa” migration and after separation of the Caucasian and the Asian populations (Figure 4). Further, the fact that SNP285C is found at a low frequency (1.6%) among Finnish habitants, is interesting. The genetic heritage of the Finns is incompletely understood; while their language, similar to that of the Lapps, Estonians and Hungarians belongs to the family of Finno-Uralic languages [38], there is evidence suggesting a more complex genetic background of the population [39], including Y-chromosome migration from Asia [40]. Notably, European migration started out from a few “sanctuaries” at the end of the last ice age about 10.000 years ago. While the first Finnish settlements can be dated around that time [41], there is evidence suggesting the Finnish population may have gone through a “bottleneck” 2-4000 years ago [42, 43], and the Finnish population is characterized by a spectrum of recessive diseases consistent with genetic isolation [41, 44]. Thus, the finding of a low incidence of the SNP285C in the Finnish population may have several explanations: It may have been absent in the original population and come along through subsequent immigration; thus, there is evidence for a second major immigration about 2000 years ago [44]. Further, as the Finnish subjects analysed so far, were collected from the Helsinki area, the polymorphism may also have been brought in through more recent immigrants from other European countries like Sweden. Both hypothesis implicates SNP285C may have arisen after separation of the Finnish ancestors from the main immigrants into Western Europe. In contrast, the possibility exist that SNP285C occurred at an incidence of about 1.6% among Europeans in general 2-4000 years ago. Thereafter, it has expanded through selection pressure in the general European population but, for reasons unexplained, not within the Finnish population.

To possibly date the origin of the 285C polymorphism, there is a need to study the distribution of the SNP285C across additional European populations, including Estonians and Hungarians in particular, as well as other European “isolated” populations that have gone through genetic “bottle-necks”, like the Basques and Sardinians [45]. Whatever its exact age may be, its spread in Western European populations strongly suggests a positive selection pressure. As argued with respect to SNP309G, the effect of SNP285C on cancer risk may probably not explain evolutionary selection for this variant in Caucasians.

While there seems to be a positive selection for SNP309G in the Caucasian as well as Asian ethnic groups, it is remarkable with a subsequent selection for the SNP285C, antagonizing SNP309G, among Caucasians. However, the Western European population has passed through distinct evolutionary “bottle-necks” during history. At the end of the ice age, only a limited number of Europeans survived in certain “refugees” like in Northern Spain, Ukraine and Moldovia [46]. As for more recent times, the black plague eradicated between one half and two-thirds of the European population between 1346-50. Further, during the 10-12.000 years passing since the last ice age, there has been severe climate alterations, causing substantial changes in the living conditions in general. It is not unlikely that fine-tuning of MDM2 levels, like the balance between SNP309 and SNP285 status, may have provided survival advantages under some stages of these shifting conditions.

Understanding the mechanisms of evolutionary selection for *MDM2* SNP309G as well as SNP285C may add important information to our understanding of the role of this complicated gene in human biology.
